# Sol-Gel Zinc Oxide Humidity Sensors Integrated with a Ring Oscillator Circuit On-a-Chip

**DOI:** 10.3390/s141120360

**Published:** 2014-10-28

**Authors:** Ming-Zhi Yang, Ching-Liang Dai, Chyan-Chyi Wu

**Affiliations:** 1 Department of Mechanical Engineering, National Chung Hsing University, Taichung 402, Taiwan; E-Mail: d099061005@mail.nchu.edu.tw; 2 Department of Mechanical and Electro-Mechanical Engineering, Tamkang University, Tamsui 251, Taiwan; E-Mail: ccwu@mail.tku.edu.tw

**Keywords:** humidity sensor, ring oscillator circuit, zinc oxide nanowire

## Abstract

The study develops an integrated humidity microsensor fabricated using the commercial 0.18 μm complementary metal oxide semiconductor (CMOS) process. The integrated humidity sensor consists of a humidity sensor and a ring oscillator circuit on-a-chip. The humidity sensor is composed of a sensitive film and branch interdigitated electrodes. The sensitive film is zinc oxide prepared by sol-gel method. After completion of the CMOS process, the sensor requires a post-process to remove the sacrificial oxide layer and to coat the zinc oxide film on the interdigitated electrodes. The capacitance of the sensor changes when the sensitive film adsorbs water vapor. The circuit is used to convert the capacitance of the humidity sensor into the oscillation frequency output. Experimental results show that the output frequency of the sensor changes from 84.3 to 73.4 MHz at 30 °C as the humidity increases 40 to 90 %RH.

## 1. Introduction

Humidity sensors are widely used in industrial, electronic, and biomedical equipment. Conventional humidity sensors have the disadvantages of large volume and high cost. On the contrary, the advantages of humidity microsensors include small volume, low cost, high performance and easy mass-production [1]. Recently, microelectromechanical system (MEMS) technology was employed to develop various microsensors, including several humidity microsensors. For instance, Wang *et al.* [2] proposed a resistive humidity sensor fabricated using the MEMS technology. The sensing material of the humidity sensor was a quaternary acrylic resin. The sensor had a humidity hysteresis of 1%–2% in the humidity range of 11–98 %RH. Kim *et al.* [3] also used MEMS technology to manufacture a humidity microsensor that consisted of a top electrode with branch structure, a bottom electrode and a sensing layer. The sensing layer was polyimide that was etched by O_2_ plasma to increase its sensitivity. Liang *et al.* [4] presented a resistive humidity microsensor made by a micromachining process. The sensor was composed of a sensitive ZnO-In_2_O_3_ film and Pt interdigitated electrodes. The sensing material of ZnO-In_2_O_3_ was deposited by radio-frequency sputtering.

Zinc oxide can be applied as a piezoelectric, gas-sensing and photoelectric material. Many studies have utilized zinc oxide as the sensitive material of humidity microsensors. For instance, Zhang *et al.* [5] fabricated a humidity microsensor on a Si substrate. The sensitive material of the sensor was zinc oxide prepared by a vapor phase method. The humidity sensor could operate at room temperature. Chang *et al.* [6] presented a humidity microsensor manufactured by MEMS technology. The sensing material of the sensor was high-density ZnO nanowires. The humidity sensor had a linear output at 80 °C. Hong *et al.* [7] employed MEMS technology to develop a surface acoustic wave humidity microsensor. The sensitive material was ZnO nanorods synthesized by a hydrothermal method. The sensitivity of the sensor was 9.4 kHz/%RH at 25 °C. Tsai *et al.* [8] used a hydrothermal growth method to make a ZnO nanosheet humidity microsensor. The sensor had a good response at room temperature, and the humidity hysteresis was less than 5%. Kiasari *et al.* [9] proposed a resistive humidity microsensor, and its sensitive material was zinc oxide nanowires deposited by chemical vapor deposition (CVD). The ZnO structures and performances of the humidity microsensors [5–9] are summarized in Table 1. These microsensors [5–9] were not integrated with readout circuit on-a-chip. Microsensors with readout circuit on-a-chip have the benefits of low package cost, low noise, low interference and high performance [1]. In this study, we fabricate a humidity microsensor integrated with a ring oscillator circuit on-a-chip. The fabrication of ZnO sensitive film in this work is easier than those sensors [5,6,8,9], and the response time is faster than that reported by Tsai *et al.* [8] and Kiasari *et al.* [9].

The commercial CMOS process has been utilized to manufacture various microactuators and microsensors [10,11]. Microsensors made by this process can integrate with readout circuits on-a-chip [12]. The humidity microsensor with a readout circuit on-a-chip proposed by Hu *et al.* [13], was fabricated using the commercial 0.18 μm CMOS process. The humidity sensor was a resistive type, and its sensitive material was titanium dioxide. The resistance of the sensor was converted into the output voltage by the readout circuit. In this work, we use the same process to develop a capacitive humidity microsensor with a ring oscillator circuit on-a-chip. Zinc oxide is adopted as the sensitive material of the sensor, because it has good sensitivity to water vapor [5–9]. The capacitance of the sensor is converted into the oscillation frequency output by the ring oscillator circuit. The output frequency has a potential for application in wireless communication system. The sensor requires a post-process [14] to coat the sensitive ZnO material. This post-process includes etching the sacrificial oxide layer and depositing the ZnO film.

## 2. Structure of the Integrated Humidity Sensor

**Figure 1.**
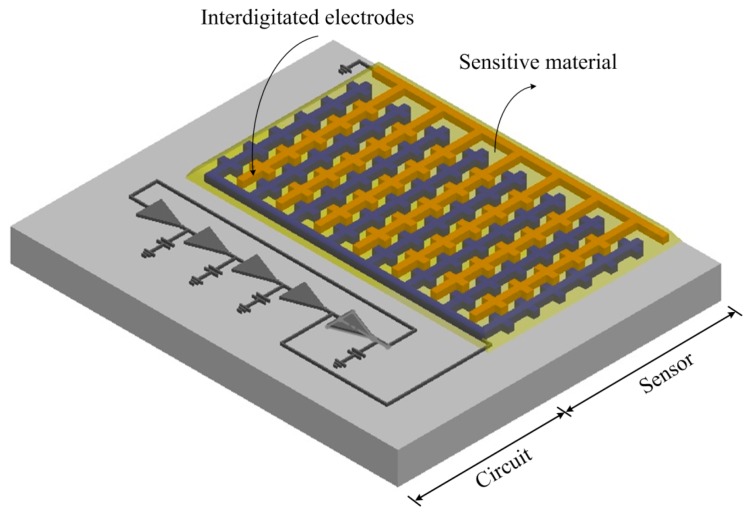
Schematic structure of the integrated humidity sensor.

Figure 1 shows the schematic structure of the integrated humidity sensor chip that contains a humidity sensor and a ring oscillator circuit. The humidity sensor is of the capacitive type. The ring oscillator circuit is used to convert the capacitance of the humidity sensor into the frequency output. The humidity sensor consists of branch interdigitated electrodes and a sensitive film. The interdigitated electrodes are constructed from the aluminum metal of the CMOS process. The length, width and thickness of the interdigitated electrodes are 320 μm, 10 μm and 6 μm, respectively. The gap between the electrodes is 10 μm. The area of the chip is about 1 mm^2^. The sensitive film of the sensor is zinc oxide, and the film is coated on the interdigitated electrodes. When the sensitive film absorbs or desorbs humidity vapor, the sensor produces a variation in capacitance.

**Figure 2.**
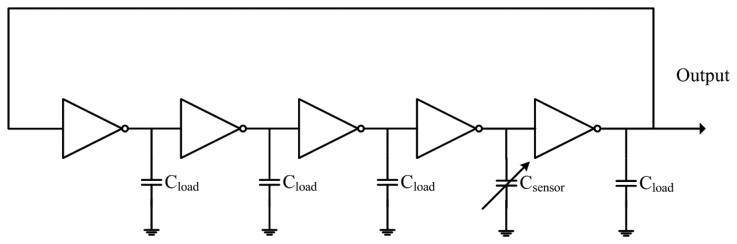
Ring oscillator circuit for the humidity sensor.

**Figure 3.**
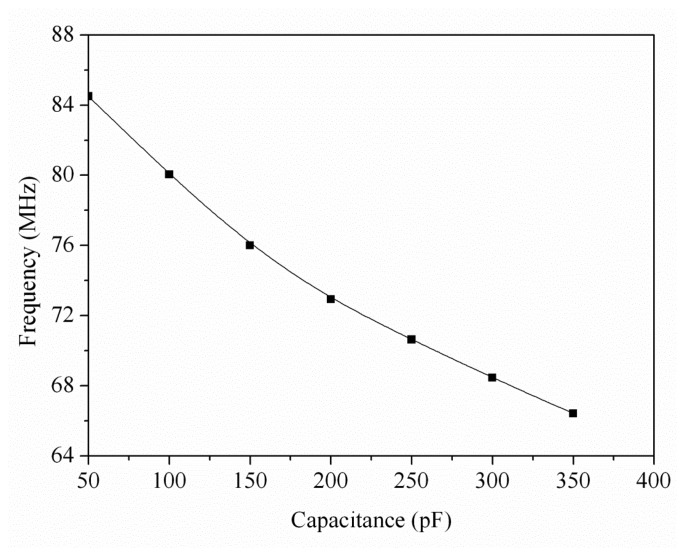
Simulation results of output frequency for the ring oscillator circuit.

Figure 2 illustrates the five-stage ring oscillator circuit for the humidity sensor. The five-stage ring oscillator circuit converts the capacitance variation of the humidity sensor into the output frequency. The oscillation frequency *f_sensor_* of the ring oscillator circuit is given by Equation (1) [15,16]:

(1) $$f_{\text{sensor}}=\frac{1}{8\tau_{\text{inv}}+2\tau_{\text{sensor}}}=\frac{1}{\frac{8C_{\text{load}}\Delta V}{I_{\text{ave}}}+\frac{2C_{\text{sensor}}\Delta V}{I_{\text{ave}}}}$$

where *τ_sensor_* the delay time associated with the humidity sensor; *τ_inv_* is the delay time associated with the inverters; *C_sensor_* is the humidity sensor capacitance; *C_load_* is the load capacitance; and *ΔV* and *I_ave_* are the threshold voltage and average current, respectively. According to Equation (1), the oscillation frequency of the ring oscillator circuit changes as the capacitance of the humidity sensor varies. The professional circuit simulation software, HSPICE (Synopsys Inc., Mountain, CA, USA), is utilized to simulate the output frequency of the ring oscillator circuit. In the simulation, the bias voltage of 3 V was adopted and the load capacitance was 0.5 pF. The capacitance of the humidity sensor changed from 50 to 350 pF. Figure 3 shows the simulated results of the output frequency for the ring oscillator circuit. The results showed that the oscillation frequency of the circuit changed from 87 to 72 MHz as the capacitance increased from 50 to 350 pF.

## 3. Preparation of Zinc Oxide

The sensitive material of zinc oxide was prepared by the sol-gel method. The preparation steps were as follows [17]: zinc acetate (0.11 g) was dissolved in *iso*-propanol (100 mL), and the mixture was denoted solution A. Sodium hydroxide (0.5 g) and poly(vinyl pyrrolidone) (2 g) were added to *iso*-propanol (50 mL), and the misture was denoted solution B. Solution B and hexamethylenetetramine (0.7 g) were added to solution A with vigorous stirring at 75 °C for 2 h. The mixture was transferred into a teflon-lined stainless steel autoclave, sealed and maintained at 120 °C for 12 h. After the reaction, the resulting products were filtered, and washed with deionized water and ethanol. Finally, the zinc oxide film was coated on the substrate, followed by calcination at 350 °C for 2 h.

**Figure 4.**
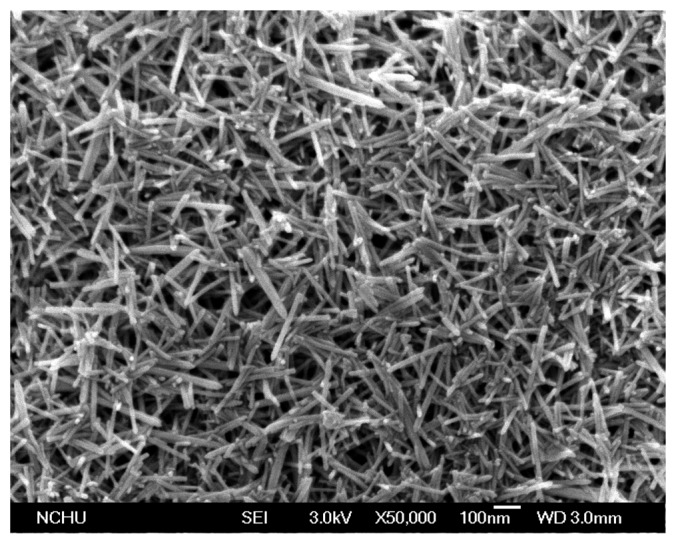
SEM image of the zinc oxide film.

**Figure 5.**
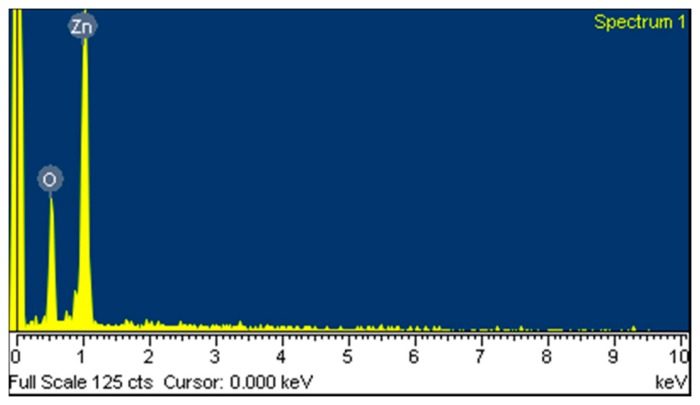
Elements of the zinc oxide film measured by EDS.

The surface morphology of the zinc oxide film was measured by scanning electron microscopy (SEM, JSM-6700F, JEOL, Tokyo, Japan). Figure 4 shows a SEM image of the zinc oxide film. The zinc oxide film is nanowire structures that can increase its sensitivity due to have a large surface area. The composition of the zinc oxide film was tested by the energy dispersive spectrometer (EDS). Figure 5 shows the EDS analysis of the zinc oxide. The main elements of the film are zinc and oxygen. The results depicted that the zinc oxide film consisted of zinc 75.97 wt% and oxygen 24.03 wt%.

## 4. Fabrication of the Integrated Humidity Sensor

**Figure 6.**
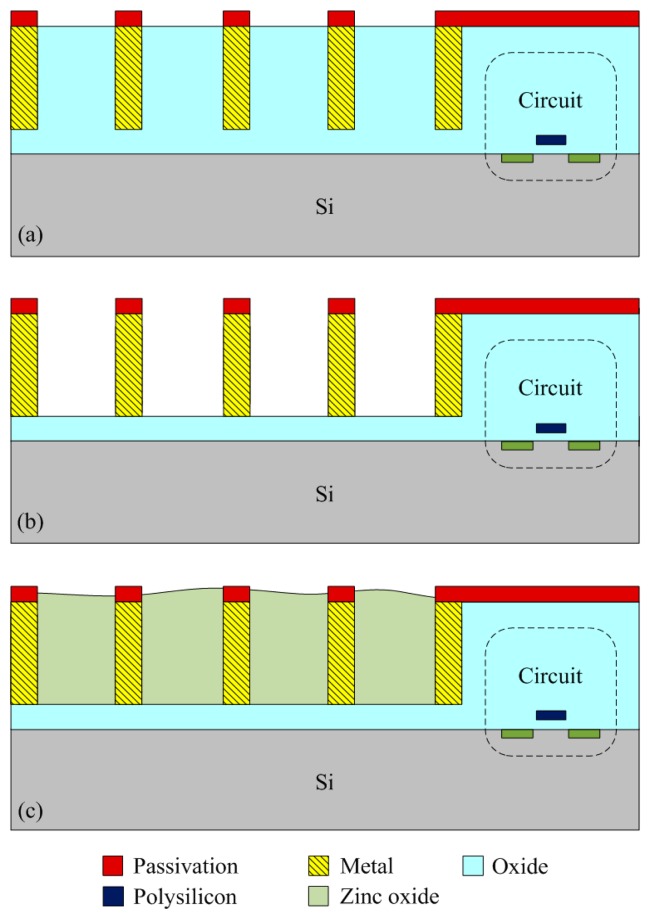
Process flow of the integrated humidity sensor, (**a**) after the CMOS process; (**b**) etching the sacrificial oxide layer; (**c**) coating the sensitive ZnO film.

The integrated humidity sensor chip was manufacture using the commercial 0.18 μm CMOS process of TSMC (Taiwan Semiconductor Manufacturing Company, Taipei, Taiwan). Figure 6 shows the process flow of the integrated humidity sensor.

**Figure 7.**
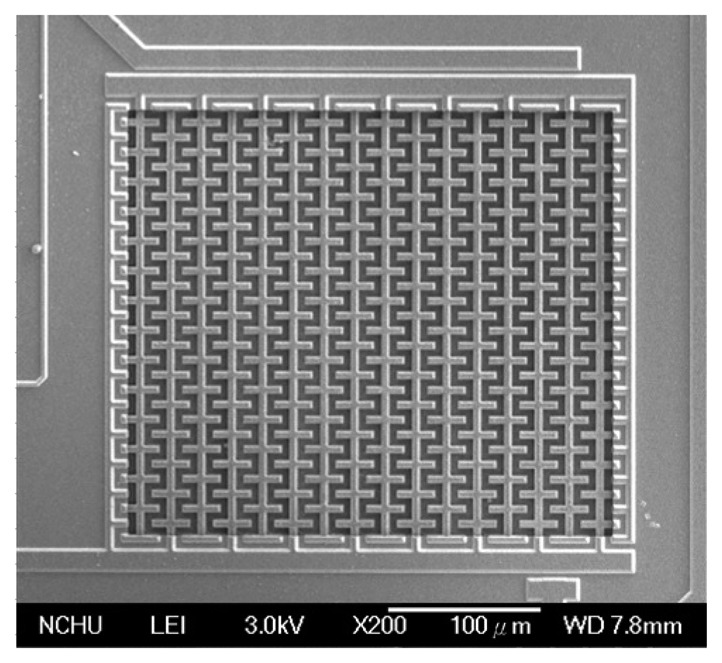
SEM image of the humidity sensor.

**Figure 8.**
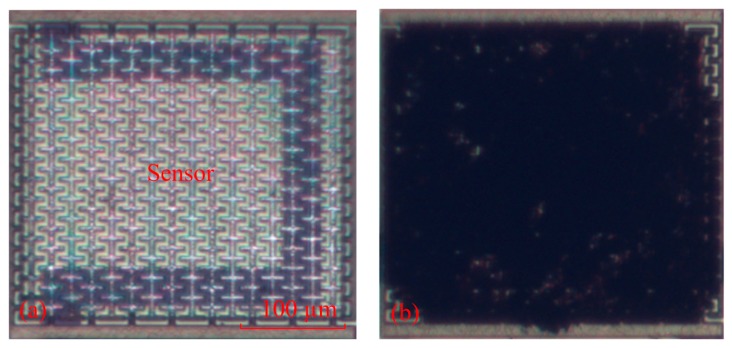
Optical image of the humidity sensor: (**a**) before the post-process; (**b**) after the post-process.

Figure 6a illustrates the cross-sectional view of the integrated humidity sensor after the CMOS process. The material of the interdigitated electrodes was aluminum metal. The silicon dioxide between the interdigitated electrodes was the sacrificial layer. After completion of the CMOS process, the humidity sensor required a post-process to etch the sacrificial layer and to coat the sensitive film of zinc oxide on the interdigitated electrodes. Figure 6b indicates that the sacrificial layer of silicon dioxide is etched. The sacrificial oxide layer was removed using the buffer etch oxide (BOE) etchant [18,19], and the interdigitated electrodes were exposed. The silicon dioxide etch must to be timed in order to avoid over-etching. The etching rate of BOE for silicon dioxide was about 960 Å/min [20]. The etching time of the silicon dioxide was 45 min. Figure 7 shows a SEM image of the humidity sensor after the etching process. The image depicts that the interdigitated electrodes of the sensor were exposed. Figure 6c shows that the sensitive material of ZnO is deposited. A precision-control micro-dropper was utilized to drop the sensitive material of ZnO onto the interdigitated electrodes. Finally, the zinc oxide was sintered in air at 350 °C for 2 h. Figure 8a shows an optical image of the humidity sensor before the post-process. Figure 8b is an optical image of the humidity sensor after the post-process.

## 5. Results and Discussion

A spectrum analyzer, a test chamber (HRMB-80, Taichy Technology Ltd., New Taipei, Taiwan) and an LCR meter were employed to test the characteristics of the integrated humidity sensor. The capacitance variation of the humidity sensor was measured by the LCR meter. The output frequency of the humidity sensor was recorded by the spectrum analyzer. The humidity and temperature of the test chamber could be tuned. The test chamber could supply a humidity range of 30–95 %RH and a temperature range of 25–100 °C.

**Figure 9.**
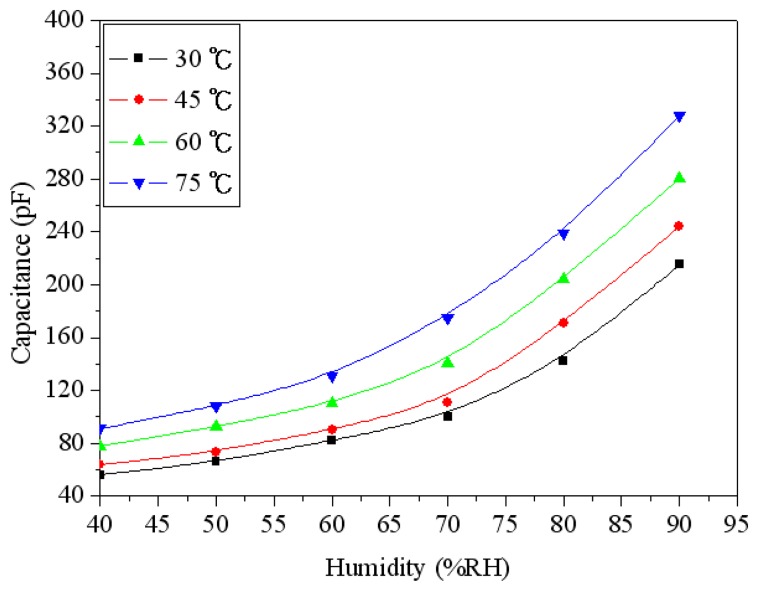
Measured capacitance of the humidity sensor at different temperatures.

**Figure 10.**
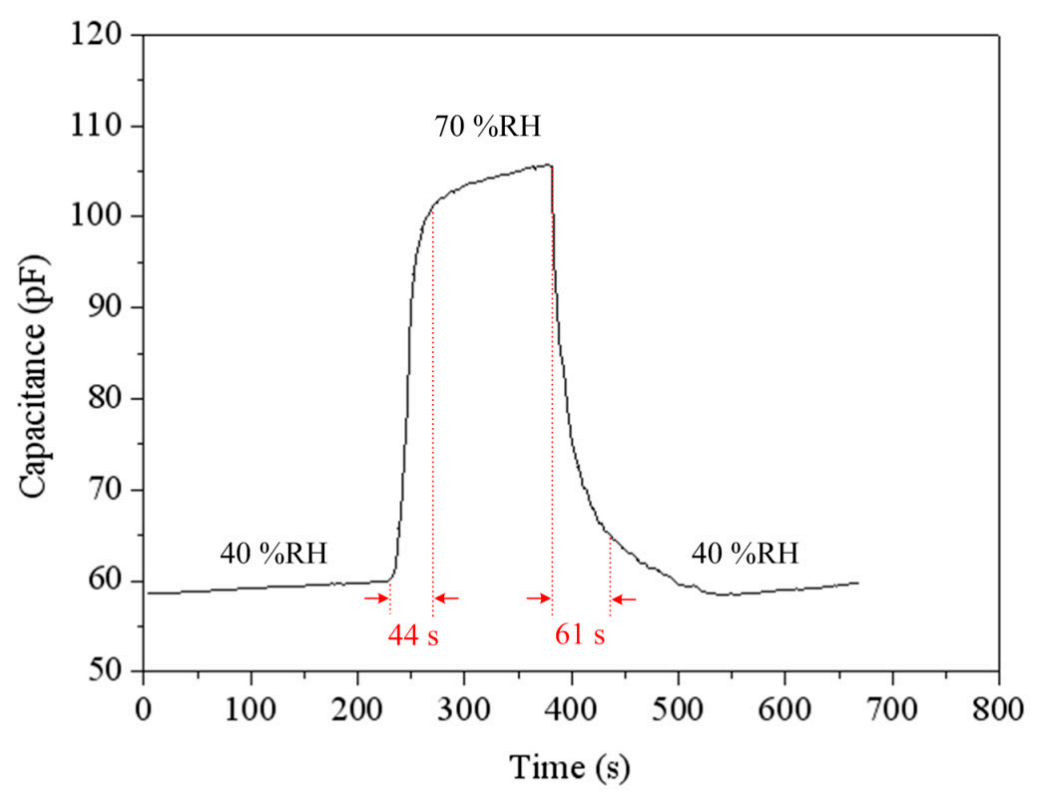
Response and recovery characteristic of the humidity sensor.

To understand the capacitance variation of the humidity sensor, the sensor without the ring oscillator circuit was tested under different humidity. The humidity sensor without the circuit was set in the test chamber. The test chamber provided different humidity to the sensor, and the LCR meter recorded the capacitance variation of the sensor. Figure 9 shows the capacitance variation of the humidity sensor at different temperatures. The measured results showed that the capacitance of the sensor was increased from 56 pF to 215 pF as the humidity changed from 40 to 90 %RH at 30 °C. When the temperature increased to 75 °C, the capacitance changed from 91 pF to 328 pF as the humidity varied from 40 to 90 %RH. Figure 10 shows the response and recovery characteristics of the humidity sensor at 30 °C. The results showed that the humidity sensor had a response time of 44 s and a recovery time of 61 s.

**Figure 11.**
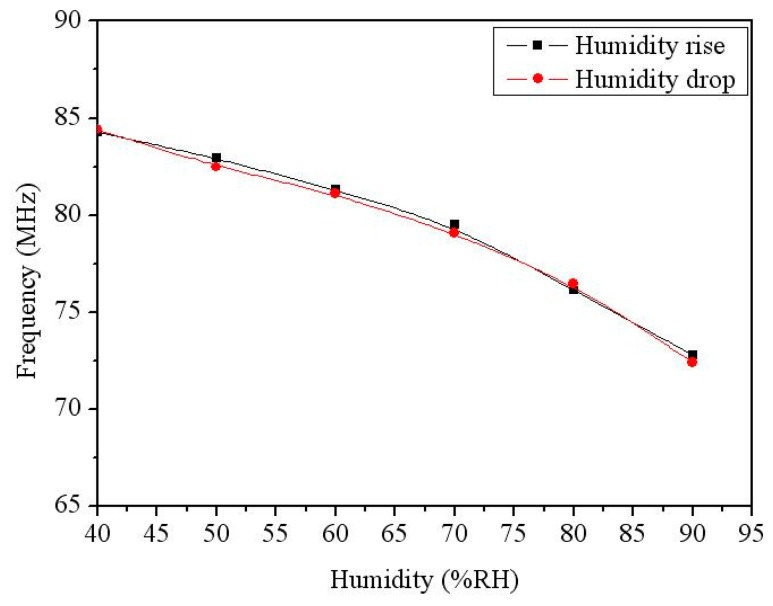
Measured output frequency of the integrated humidity sensor at 30 °C.

The output frequency of the humidity sensor with the ring oscillator circuit was measured. The ring oscillator circuit converted the capacitance variation of the humidity sensor into the oscillation frequency output. The sensor with the circuit was set in the test chamber. The power supply provided a bias voltage of 3 V to the circuit. The spectrum analyzer detected the output frequency of the sensor. Figure 11 shows the output frequency of the integrated humidity sensor. In this measurement, the temperature was kept at 30 °C, and the humidity changed from 40 to 90 %RH. The measured results revealed the output frequency of the sensor varied from 84.3 to 73.4 MHz as the humidity increased 40 to 90 %RH, and the humidity hysteresis was less than 1%.

**Figure 12.**
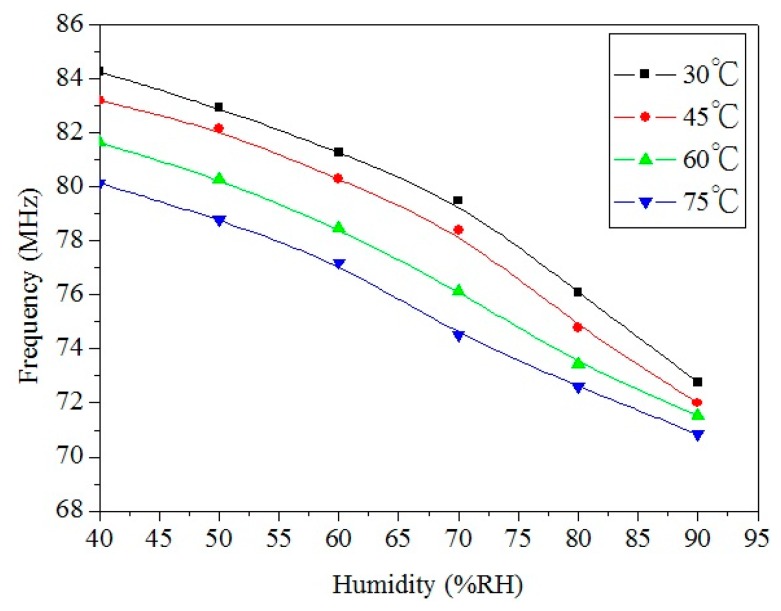
Measured output frequency of the humidity sensor at different temperatures.

To characterize the influence of temperature, the integrated humidity sensor was tested under different temperatures. Figure 12 shows the measured output frequency of the humidity sensor at different temperatures. The results showed that the output frequency of the sensor decreased from 84.3 MHz at 30 °C to 80 MHz at 75 °C when the humidity was 40 %RH, and the output frequency also decreased from 73.4 MHz at 30 °C to 71 MHz at 75 °C as the humidity was 90 %RH. Thereby, when the temperature increased, the output frequency of the sensor reduced.

Hu *et al.* [13] reported a humidity sensor manufactured by the CMOS process. The sensitive film was nanoparticle titanium dioxide. The response and recovery times of the humidity sensor were 58 s and 65 s, respectively. The sensitive film in this work was zinc oxide that has nanowire and porous structures. Thereby, the response and recovery times in this work are faster than that of Hu [13]. Dai [21] utilized the commercial CMOS process to fabricate a humidity sensor with three-stage ring oscillator circuit. The sensing material of the sensor was polyimide and the sensitivity was 14.5 kHz/%RH at 25 °C. This work proposed a humidity sensor with five-stage ring oscillator circuit and the sensitivity was 230 kHz/%RH at 30 °C. A comparison to Dai [21], the sensitivity of the sensor in this work exceeds that of Dai [21]. André *et al.* [22] presented an airflow sensor with a five-stage ring oscillator circuit. The oscillation frequency of the ring oscillator was 270 kHz at 0.6 V bias voltage, and the power consumption was about 1 μW. The oscillation frequency of the ring oscillator in this work was 84.3 MHz at 3 V bias voltage, and the power consumption was 20 mW. The power consumption in this work is higher than that of André *et al.* [22].

## 6. Conclusions

An integrated humidity sensor has been fabricated using the commercial 0.18 μm CMOS process and a post-process. The integrated humidity sensor contained a humidity sensor and a ring oscillator circuit. The humidity sensor was a capacitive type. The sensor generated a change in capacitance when it sensed water vapor. The ring oscillator circuit converted the capacitance variation of the sensor into the output frequency. The humidity sensor consisted of branch interdigitated electrodes and a sensitive film. The sensitive film was zinc oxide that prepared by the sol-gel method. The post-process included a wet etching to remove the sacrificial oxide layer and a zinc oxide film to coat on the interdigitated electrodes. The experimental results revealed that the output frequency of the sensor changed from 84.3 to 73.4 MHz at 30 °C when the humidity increased 40 to 90 %RH.

## Figures and Tables

**Table 1.** ZnO structures and performances of the humidity sensors in [5–9].

**Reference**	**ZnO Fabrication**	**ZnO Structure**	**Response/Recovery Time (s)**	**Humidity Range (%RH)**
[5]	Vapor phase transport	Nanorod & nanowire	3/10	12–97
[6]	Sputtering	Nanowire	−	25–90
[7]	Sol-gel	Nanorod	−	10–90
[8]	Sputtering	Nanosheet	600/3	12–96
[9]	CVD	Nanowire	60/3	0–60
This work	Sol-gel	Nanowire	44/61	40–90
